# Elevated apolipoprotein C3 heightens atherosclerosis risk by mediating arterial accumulation of free cholesterol and local inflammation in diabetes

**DOI:** 10.21203/rs.3.rs-6979508/v1

**Published:** 2025-07-16

**Authors:** Jenny E. Kanter, Cheng-Chieh Hsu, Farah Kramer, Baohai Shao, Tomas Vaisar, Laura J. den Hartigh, Abigail Reed, Jason Luo, Alan Tran, Jingjing Tang, Henry Mangalapalli, Jocelyn Cervantes, Masami Shimizu-Albergine, Peter D. Reaven, Juraj Koska, Majken K. Jensen, Brandon S.J. Davies, Edward A. Fisher, Nicholas O. Davidson, Nathan O. Stitziel, Adam E. Mullick, Ira J. Goldberg, Karin E. Bornfeldt

**Affiliations:** 1 Department of Medicine, Division of Metabolism, Endocrinology and Nutrition, University of Washington, Seattle, WA 98109; 2 UW Medicine Diabetes Institute, University of Washington, Seattle, WA 98109; 3 Department of Laboratory Medicine and Pathology, University of Washington, Seattle, WA 98109; 4 Phoenix VA Health Care System, Phoenix, AZ 85012; 5 Department of Public Health, University of Copenhagen, 1353 Copenhagen, Denmark; 6 Department of Biochemistry and Molecular Biology, University of Iowa, Iowa City, IA 52242; 7 Fraternal Order of Eagles Diabetes Research Center, University of Iowa, Iowa City, IA 52242,; 8 Holman Division of Endocrinology, Department of Medicine, New York University Grossman School of Medicine, New York, NY 10016; 9 Diabetes, and Metabolism, and Division of Cardiology, Department of Medicine, New York University Grossman School of Medicine, New York, NY 10016; 10 Department of Medicine, Division of Gastroenterology, Washington University School of Medicine, Saint Louis, MO 63110; 11 Cardiovascular Division, Washington University School of Medicine, Saint Louis, MO 63110; 12 Department of Genetics, Washington University School of Medicine, Saint Louis, MO 63110; 13 Ionis Pharmaceuticals, Inc., Carlsbad, CA 92010, USA.

## Abstract

Cardiovascular outcome trials are being considered for therapeutics that silence apolipoprotein C3 (APOC3) or angiopoietin-like 3 (ANGPTL3) because of their abilities to lower triglyceride-rich lipoproteins (TRLs) and their remnants in individuals with increased cardiovascular disease (CVD) risk^1–4^. Here we demonstrate that plasma APOC3 predicts CVD events in individuals with diabetes more strongly than in those without diabetes. Accordingly, plasma APOC3 levels are elevated, clearance of TRLs/remnants is slowed, and plasma TRL remnants are increased in two mouse models of diabetes-accelerated atherosclerosis. Silencing mouse APOC3 by a liver-targeted antisense oligonucleotide lowers both cholesterol and triglycerides carried by TRL/remnants and LDL and prevents aortic free cholesterol accumulation in diabetes, while ANGPTL3 silencing reduces triglycerides. Single-cell RNA-sequencing revealed that APOC3 silencing prevents a majority of diabetes-induced pathways in macrophages, endothelial cells, and smooth muscle cells, with inflammation as a major predicted upstream regulator, adding promise to APOC3 as a CVD target in diabetes.

The link between *APOC3* loss-of-function variants and triglyceride lowering with apparent CVD protection was discovered in 2008^5^ and confirmed by subsequent studies^6,7^, surging an interest in APOC3 as a target for prevention of residual CVD risk. Like APOC3, ANGPTL3 has been linked to CVD risk^8,9^. Both APOC3 and ANGPTL3 increase plasma triglyceride levels by inhibiting lipoprotein lipase, but these circulating proteins also have additional distinct effects. For example, APOC3 prevents hepatic uptake of TRLs and their remnants by interfering with lipoprotein uptake through LDL family receptors (LDLR and LDLR-related protein 1; LRP1).^10^ ANGPTL3 inhibits endothelial lipase^11,12^ and promotes hepatic TRL secretion^13^. Thus, therapeutic silencing of hepatic expression of APOC3 or ANGPTL3 lowers levels of TRL particles and their smaller remnant lipoprotein particles (RLPs) generated by partial TRL lipolysis^14^. TRL particles are believed to be more atherogenic than LDL particles^15^, as they, per particle, may deposit more cholesterol^16^ and create more vascular inflammation^17^.

Residual CVD risk in individuals on LDL-lowering medications is linked to elevated levels of TRLs, especially in those with diabetes^16,18^. Although hyperglycemia has historically received the most attention for causing the increased clinical CVD risk associated with diabetes, altered lipoprotein metabolism^19^ clearly contributes to CVD risk in diabetes^20^. Indeed, substantial CVD risk associated with non-traditional CVD risk factors related to TRL metabolism, such as APOC3, remains in individuals with type 1 or type 2 diabetes^21–24^. Moreover, mouse models of type 1 and type 2 diabetes demonstrate that APOC3 is a causal mediator of diabetes-associated atherosclerosis^21,25^.

To investigate if APOC3 is a stronger predictor of CVD risk in individuals with diabetes than in those without diabetes, we analyzed the association of baseline plasma APOC3 measured *before* CVD events in 5,743 participants in the Multiethnic Study of Atherosclerosis (MESA), 4,187 of whom had normal fasting glucose levels (below 100 mg/dL), 822 of whom had elevated fasting glucose (100–125 mg/dL), and 724 of whom had type 2 diabetes (fasting glucose ≥ 126 mg/dL). Incident CVD events were defined as myocardial infarction, resuscitated cardiac arrest, or stroke. This analysis showed that baseline plasma APOC3 levels associate with CVD risk more strongly in individuals with type 2 diabetes than in those with impaired fasting glucose or without diabetes (Table 1, Extended Data Fig. 1a). Moreover, in the 724 participants with diabetes, baseline APOC3 predicted incident CVD independently of traditional CVD risk factors, including LDL-cholesterol, HDL-cholesterol, and triglycerides (Extended Data Fig. 1b). These results suggest that APOC3 silencing might be more effective in preventing CVD risk in individuals with diabetes than in those without diabetes.

Because APOC3 and ANGPTL3 are both under consideration as therapeutic targets for CVD prevention^26^, we next contrasted the effects of silencing hepatic APOC3 versus ANGPTL3 on diabetic dyslipidemia, arterial lipid accumulation, and initiation of lesions of atherosclerosis. We used two LDLR-deficient mouse models of diabetes-accelerated atherosclerosis: the streptozotocin (STZ)-induced model and the transgenic lymphocytic choriomeningitis virus (LCMV)-induced autoimmune diabetes model, in which β-cell destruction is mediated by CD8^+^ T cell attack. Hepatic APOC3 and ANGPTL3 were silenced using N-acetylgalactosamine (GalNAc)-modified antisense oligonucleotides (ASOs). Littermate controls received a control ASO (cASO).

Both diabetes models exhibited hyperglycemia and a striking time-dependent aortic neutral lipid accumulation, measured as *en face* Sudan IV-positive area, compared with non-diabetic littermates (Fig. 1a-b, Extended Data Fig. 2a-b). The aortic lipid accumulation was unlikely to be explained solely by elevated plasma levels of total cholesterol or total triglycerides because while plasma lipids were higher in the LCMV model of diabetes, plasma lipid levels were not significantly elevated in the STZ diabetes model (Extended Data Fig. 2c-f). The atherosclerotic lesions were fatty streaks containing macrophages (Mac-2^+^ cells) and intimal/subintimal accumulation of APOB, present in TRLs/RLPs and LDL (Extended Data Fig. 2g). Thus, diabetes results in a marked and time-dependent aortic accumulation of APOB lipoproteins through a process that is not due just to elevated plasma lipid levels. Possible explanations for this marked neutral lipid accumulation include increased arterial uptake of APOB-containing lipoproteins or selective subpopulations (LDL, TRLs, or RLPs) due to increased lipoprotein particle plasma concentrations or increased endothelial uptake/transcytosis, increased trapping and retention of APOB lipoproteins in the intima due to changes in the extracellular matrix, or insufficient clearance of arterial APOB lipoproteins.

We next used calibrated differential ion mobility analysis^27^ to directly measure circulating TRL/RLP and LDL particle concentrations and sizes. Both diabetes models exhibited increased plasma concentrations of TRL/RLP and LDL particles (Extended Data Fig. 3a-d). Particularly, diabetes increased TRLs in the small to medium (23–36 nm diameter) range, strongly indicating that these particles are remnants (RLPs). Experiments in which diabetic and non-diabetic mice were injected with radiolabeled- or DiI-labeled TRLs demonstrated that the increased plasma concentrations of RLP particles were explained by reduced TRL clearance rates, a finding consistent in both diabetes models (Extended Data Fig. 3e-g). Consistently, both diabetes models had elevated plasma APOC3 levels (Extended Data Fig. 3h-i), consistent with our previous studies^21,25,28^.

Because the more severely diabetic autoimmune model more closely mimics human diabetic dyslipidemia, which exists both in individuals with poorly controlled type 1 diabetes and in individuals with type 2 diabetes^19^, we selected this model for hepatic APOC3- and ANGPTL3-silencing. The APOC3 ASO effectively lowered the elevated plasma APOC3 and hepatic *Apoc3* mRNA in diabetic mice without affecting blood glucose levels, and also lowered APOC3 in non-diabetic littermates (Fig. 1c-d, Extended Data Fig. 4).

APOC3 ASO prevented the elevated plasma triglycerides, cholesterol, TRL particle concentration (as assessed by APOB content in chylomicron-depleted samples), TRL-cholesterol, and TRL-triglycerides in diabetic mice (Fig. 1e-k), consistent with augmented TRL catabolism due to APOC3 silencing^14^. The effects were also observed in non-diabetic mice, albeit generally to a lesser extent. Furthermore, APOC3 ASO lowered LDL particle concentration, LDL-cholesterol, and LDL-triglycerides in diabetic mice (Fig. 1l-n) without significantly altering these LDL metrics in non-diabetic mice (Fig. 1l-n). APOC3 ASO also altered the LDL and TRL/RLP proteome in a manner characteristic of increased lipoprotein particle clearance, with reduced levels of APOCs and APOE, indicating that APOC3 ASO normalized the slowed RLP clearance in diabetes (Extended Data Fig. 5a-k). Similar differences were observed by diabetes in the LDL density range, suggesting the presence of RLPs in this fraction (Extended Data Fig. 5l-v)

ANGPTL3 ASO lowered hepatic *Angptl3* but not *Apoc3* mRNA levels and markedly lowered plasma ANGPTL3 levels in diabetic mice, although the APOC3 ASO was more effective at silencing its target (Extended Data Fig. 4a-d). The elevated plasma ANGPTL3 in the autoimmune diabetes model, but not in the STZ diabetes model tracked with the more severe hyperlipidemia in that model (Extended Data Fig. 3j, 4d). The main effect of ANGPTL3 ASO on plasma lipids was due to increased triglyceride lipolysis, perhaps in combination with reduced hepatic TRL secretion rate^13^, as plasma triglycerides, TRL- and LDL-triglycerides were dramatically suppressed (Extended Data Fig. 4e-k) without changes in the TRL/RLP or LDL proteomes (Extended Data Fig. 5a-v). The small cholesterol-lowering effect was likely due in part to LDL particle lowering (Extended Data Fig. 4l-n). Neither ASO had detrimental effects on hepatic lipid content or blood glucose (Extended Data Fig. 4o-q).

Together, these results support the conclusion that hepatic APOC3 silencing may be more beneficial in the setting of diabetes as it promotes hepatic clearance of smaller cholesterol-containing RLP particles in addition to triglyceride-lowering, while the main effect of ANGPTL3 silencing in diabetes is to lower triglycerides.

Given the different effects of APOC3 and ANGPTL3 silencing on circulating APOB-lipoprotein cholesterol in diabetic mice, we next asked how APOC3 and ANGPTL3 silencing affects aortic lipid accumulation. Diabetes caused a marked increase in aortic Sudan IV staining, which was explained by significant increases in total cholesterol, cholesteryl esters, and triglycerides (Fig. 2a-d). While APOC3 and ANGPTL3 silencing both exerted modest protective effects on aortic Sudan IV-positive neutral lipid accumulation and fatty streak brachiocephalic lesion area in diabetic mice (Fig. 2a, Extended Data Fig. 6a-c), only APOC3 silencing reduced aortic total and free cholesterol in diabetic mice. Strikingly, APOC3 ASO completely normalized the elevated aortic free cholesterol in diabetic mice. Neither APOC3 nor ANGPTL3 silencing lowered aortic cholesteryl ester accumulation (Fig. 2b-d, Extended Data Fig. 6d-f). Conversely, only ANGPTL3 significantly lowered the elevated aortic triglyceride content (Fig. 2e, Extended Data Fig. 6g). These results raise the interesting possibility that silencing of either APOC3 or ANGPTL3 results in modest protective effects on early lesion size in diabetic mice, but that APOC3 silencing has additional beneficial effects in preventing free cholesterol accumulation, due to its ability to lower TRL/RLP- and LDL-cholesterol levels. It should be noted that the beneficial effect of hepatic APOC3 silencing on early lesions of atherosclerosis in the present study was more modest than in our previous study in which a non-liver-targeted APOC3 ASO was used^21^. It is possible that this difference could be explained by differences in dosing, off-target effects by the non-liver-targeted APOC3 ASO, or by APOC3 produced by the intestine, contributing to atherosclerosis.

To gain a deeper understanding of how hepatic APOC3 silencing affects the lesioned artery in diabetes, single-cell RNA-sequencing (scRNA-seq) was performed on aortic arches from the two mouse models of diabetes and diabetic mice treated with the APOC3 GalNAc ASO (Fig. 3a-c, Extended Data Fig. 7a-f; Supplemental Excel 1–2). Each cell cluster was annotated against published data^29^. Smooth muscle cells (SMCs) comprised the largest cluster, followed by fibroblasts, while endothelial cells (ECs) and leukocytes contributed <4% each. There were no consistent differences in the relative number of cells in the identified populations in the two diabetes models (Extended Data Fig. 7b-c).

Reclustering of the leukocytes revealed the most predominant cluster as resident macrophages characterized by high expression of *Lyve1* (Fig. 3d), reflecting the previously described TLF Cd209^lo^ population^30^. There were no consistent differences in the relative number of cells in the leukocyte subclusters in the two diabetes models (Extended Data Fig. 7e-f). The resident macrophage cluster was the most abundant cluster in both models, showing an increase in relative abundance in the STZ diabetes model and a decrease in the LCMV model. To confirm the scRNA-seq data, we selected to verify the increased abundance of LYVE1-positive macrophages in STZ diabetic mice by flow cytometric analyses of digested lesioned aortas. While there were no differences in total CD11B/F4/80 macrophages in diabetic versus non-diabetic mice, diabetic mice exhibited more LYVE1^+^ macrophages (Extended Data Fig. 8a-d). Importantly, immunofluorescence showed strong intimal LYVE1 immunoreactivity overlapping with the pan-macrophage marker Mac-2 (Extended Data Fig. 7g), demonstrating that the resident macrophage cluster is intimal rather than adventitial.

Ingenuity pathway analysis (IPA; Supplemental Excel 3) was used to compare leukocyte clusters across the two diabetes models and in diabetic mice with hepatic APOC3 silencing. Using differentially expressed genes (DEGs) with adjusted p-values <0.05 and a Z-score cutoff of 2 in both models of diabetes compared to their non-diabetic controls and regulation in the opposite direction in APOC3 ASO-treated mice vs cASO-treated diabetic littermates revealed that diabetes enriched 19 pathways in the resident macrophage cluster over their non-diabetic controls. Of these 19 pathways, 11 were prevented in diabetic mice treated with APOC3 ASO (Fig. 3e). The top enriched pathway in diabetic mice strongly prevented by APOC3 silencing was neutrophil degranulation, but a pathway related to lipoprotein metabolism, driven by lipid-related genes including *Abca1*, *Abcg1*, *Apoe,* and *Soat1* was also enriched by diabetes and prevented by APOC3 silencing (Fig. 3e). Consistently, upstream regulator analysis identified the top diabetes-induced regulators prevented by APOC3 ASO as responses to PPARγ and the LXRα/β agonist GW3965, master regulators of lipid metabolism (Extended Data Fig. 9a). Together, the results indicate that diabetes enhances lipid signatures in intimal resident macrophages mediated by hepatic APOC3, but that several other diabetes-enriched pathways related to inflammation and metabolism are also prevented by APOC3 silencing.

Diabetes altered 7 pathways in ECs, 3 of which were prevented by APOC3 silencing (Fig. 3f). Like in macrophages, a major effect of diabetes was an enhancement of inflammatory signatures, with lipopolysaccharide as the major predicted positive upstream regulator (Extended Data Fig. 9b). This pathway was prevented by APOC3 silencing. One predicted upstream regulator—D-glucose—was notably enhanced with diabetes but not prevented by APOC3 ASO in ECs, consistent with the lack of effect of APOC3 silencing on blood glucose levels.

Diabetes altered 179 pathways in SMCs; of these, a full 161 were prevented by APOC3 ASO (Fig. 3g). As in ECs, diabetes enhanced inflammatory signatures, including antigen processing, integrins, and senescence signaling, which were prevented by hepatic APOC3 silencing, with response to lipopolysaccharide as a major predicted upstream regulator (Extended Data Fig. 9c). Similar to the EC data, a glucose metabolite, UDP-D-glucose, was identified as a predicted upstream regulator activated in both diabetes models and not prevented by APOC3 silencing. Likewise, in the fibroblast cluster diabetes altered 247 pathways, 235 of which were prevented by APOC3 ASO (Supplemental Excel 3).

Together, our observations raise an important question: Are the protective effects of APOC3 silencing on arterial cells in the setting of diabetes due to reduced direct effects of APOC3, caried into the lesion by lipoproteins, or to indirect effects mediated by reduced levels of circulating APOB-lipoprotein cholesterol and subsequently reduced levels of aortic free cholesterol? We have previously shown that lipid-free, but not lipid-bound, APOC3 can induce inflammasome activation in human and mouse monocytes^31^. To investigate if free APOC3 can induce transcriptomic changes, including inflammatory signatures, in arterial SMCs, we stimulated human arterial SMCs with delipidated human APOC3 at a concentration that induced inflammasome activation in human monocytes^31^. However, free APOC3 failed to induce transcriptomic responses in cultured primary aortic SMCs (Supplemental Excel 4), suggesting that the effects of APOC3 silencing are due to APOB-lipoprotein lowering rather than a to direct effects of APOC3 on SMCs. Instead, a more likely mechanism is that accumulation of free cholesterol, which has been shown to induce inflammasome activation and other inflammatory pathways in lesional cells^32^, explains the local arterial effects of APOC3. Moreover, the protective effects of APOC3 silencing on arterial inflammation are likely to be local since plasma IL-18 levels and monocytosis were unaltered (Extended Data Fig. 10a-d), consistent with studies in non-diabetic mice^33^. Thus, correcting the impaired TRL/RLP clearance by silencing APOC3 prevents a majority of diabetes-induced signatures in arterial cells and free cholesterol accumulation even when glucose levels are high (Fig. 3h).

Strengths of this study include the use of two distinct mouse models of diabetes to increase scientific rigor, and, similar to therapeutics considered for human cardiovascular outcome trials, use of liver-targeted APOC3 and ANGPTL3 GalNAc ASOs. Our findings are novel in that they show that APOC3 silencing is not only more effective in the setting of diabetes and that the harmful effects of diabetes in the artery wall are largely mediated by APOC3, but also that APOC3 is a stronger predictor of CVD risk in individuals with diabetes. Moreover, in the setting of diabetes, APOC3 silencing appears to have beneficial effects beyond those of ANGPTL3 silencing, preventing arterial free cholesterol accumulation. Our results highlight important roles for TRLs and RLPs in atherogenesis in diabetes—and APOC3 as a therapeutic target for CVD prevention in diabetes.

## Methods

### Analysis of plasma APOC3 in human participants in Multiethnic Study of Atherosclerosis (MESA).

Data used in this study were obtained from the Multi-Ethnic Study of Atherosclerosis (MESA) in accordance with their published data access policies, including an approved written proposal (https://www.mesa-nhlbi.org). The MESA study is a multicenter longitudinal study to examine factors associated with subclinical CVD and the progression from subclinical to clinical CVD in individuals aged 45 to 84 years, of non-Hispanic White, African American, Hispanic and Chinese American race/ethnicity, and without known CVD at the enrollment^34^. Diabetes was defined as fasting glucose (FG) ≥ 7.0 mmol/L (126 mg/dL) or self-reported use of hypoglycemic drugs. Impaired fasting glucose was defined as FG 5.6 – 6.9 mmol/L (100 – 125 mg/dL). Plasma concentrations of APOC3 were measured in 5,794 baseline samples by sandwich ELISA as detailed previously^35^ (see Key Reagents Supplement). CVD events were defined as the first occurrence of definite myocardial infarction, resuscitated cardiac arrest, or stroke from baseline (2000–2002) through the end of 2017. Details on CVD surveillance have been previously reported^36^. Statistical analyses were conducted using SAS v9.4 (SAS Institute, Cary, NC). APOC3 concentrations were natural log-transformed to approximate normality and scaled to a mean of 0 and a SD of 1. Linear regression was used to test the association of baseline APOC3 levels with fasting glucose categories. Cox proportional hazard regression was used to assess the association of baseline APOC3 with incident CVD events adjusted for age, sex, and race/ethnicity (Model 1), then further adjusted for use of antihypertensive medications, systolic blood pressure, and tobacco use (Model 2), then Model 2 variables and plasma LDL and HDL cholesterol (Model 3), and Model 2 variables and plasma triglycerides (Model 4). The analyses were run in the whole group and stratified by fasting glucose status. Heterogeneity between the FG groups was assessed by including an interaction term in the models.

### Mouse models of diabetes and hepatic silencing of APOC3 and ANGPTL3.

Streptozotocin (STZ) was used to induce β-cell failure in female *Ldlr*^−/−^ mice. Low-dose STZ (50 mg/kg) was injected intraperitoneally on five consecutive days. If mice did not display hyperglycemia (blood glucose >250 mg/dL) 2 weeks after STZ injections, another STZ round was administered. Citrate was injected into control littermate mice. At the onset of diabetes, the mice were fed a low-fat, semi-purified diet (10% of calories from fat, no added cholesterol)^37^ for 6, 9, or 12 weeks.

The *Ldlr*^−/−^ *Gp*^*Tg*^ mouse model of diabetes-accelerated atherosclerosis was used as a second model of diabetes. This model of autoimmune diabetes has been described previously^37^. These mice express the lymphocytic choriomeningitis virus (LCMV) glycoprotein (GP) under the control of the insulin promoter. After the virus is injected, CD8^+^ T cells kill the GP-expressing β-cells, inducing diabetes. At diabetes onset, the mice were switched to a low-fat semi-purified diet^37^ and maintained for 6 to 12 weeks.

To study the role of reducing TRLs/RLPs, APOC3 or ANGPTL3 was silenced in male *Ldlr*^−/−^ *Gp*^*Tg*^ mice using GalNAc-modified ASOs (i.p. injections, 10 mg/kg/week). The GalNAc modification is also used in the human APOC3 ASO therapeutic olezarsen, which was recently approved by the FDA as a treatment for adults living with familial chylomicronemia syndrome as an adjunct to diet. The GalNAc modification increases hepatic targeting and reduces off-target effects as compared with ASOs not modified by GalNAc. At the onset of diabetes, the mice were randomized to receive either control ASO (cASO), APOC3 ASO, or ANGPTL3 ASO and were then fed the same low-fat semi-purified diet as above for 12 weeks. Saline was injected into non-diabetic littermates. The mice were randomized into treatment groups.

### Quantifying atherosclerosis, immunostaining, and aortic lipid analysis.

Sudan IV-positive areas in the aortic arch (to 2 mm past the subclavian artery) or entire aorta carefully cleaned of perivascular adipose tissue were quantified as described^37^. To measure aortic lipids, following the aortic Sudan IV-staining, aortas were vortexed in 3.75 mL of chloroform-methanol (1:2 vol: vol), 2.5 mL of additional chloroform was added, followed by 0.5 mL of water. Following phase separation, the chloroform layer was isolated and dried down with nitrogen gas. The samples were resuspended in 100 μL of 100% isopropanol and analyzed for total cholesterol and free cholesterol using Amplex™ Red Cholesterol Assay (Invitrogen; A12216) and triglycerides using Triglyceride Assay Kit (Abcam; Ab65336). For quantifications, the signal from the Sudan IV stain was subtracted from the final values. Note that any remaining perivascular adipose tissue could contribute to the aortic triglyceride measurements.

Lesion macrophages were visualized by Mac-2 immunohistochemistry using a monoclonal rat anti-mouse Mac-2 antibody (CL8942AP; Cedarlane). Immunohistochemistry for APOB was performed using a biotinylated goat anti-APOB antibody (R&D Systems; BAF3556). The APOB signal was enhanced using Tyramide-AlexaFluor488 after incubation with streptavidin-HRP. LYVE1 was detected using an anti-LYVE1 antibody (R&D Systems; AF2125). All analyses were carried out by an investigator blinded to the experimental groups. See the Key Reagents supplement for antibody concentrations.

### Analysis of plasma lipids, APOC3, ANGPTL3, TRL clearance, and lipoprotein particles.

Blood glucose, plasma triglycerides, and cholesterol were measured throughout each of the studies (see Supplemental Table 3 for information on the number of mice/group/time-point). Blood glucose was measured from the saphenous vein blood by stick tests (OneTouch Ultra). As the glucometer does not go beyond 600 mg/dL, values above 600 mg/dL were set to 601 mg/dL. Plasma cholesterol and triglyceride levels were determined by Cholesterol Liquicolor and Triglyceride Liquicolor, respectively (Stanbio, purchased via Fisher Scientific). Cholesterol- and triglyceride-lipoprotein profiles were analyzed by fast protein liquid chromatography (FPLC), as described previously^37^. Prior to loading the samples onto the FPLC column (Superose 6 column), chylomicrons were removed by 0.45 μm-filtration. Differential ion mobility analysis (DMA) was used to determine LDL and TRL particle levels, as previously described^38,39^. Briefly, plasma was separated based on density, where LDL was isolated between 1.08 and 1.019 g/mL, and TRLs/RLPs were isolated at density <1.019 g/mL following removal of large chylomicrons (centrifugation at 10,000×g for 10 min, 4°C) before density adjustment. The 1.08 g/mL density cut-off for LDL isolation rather than the 1.063 g/mL cut-off for human LDL was used to capture a more dense particle population in mice. The LDL and TRL density fractions were subjected to calibrated DMA, using a standard consisting of known concentrations of LDL particles. APOC3, ANGPTL3, and IL-18 plasma levels were measured using ELISAs from Abcam and Invitrogen, respectively (Key Reagents Supplement).

Clearance of radiolabeled chylomicrons was measured in non-diabetic and diabetic mice fasted for 7 hours before being injected intravenously with 4.5 μL of labeled chylomicron/g body weight. Blood was collected at 1, 5, 15, and 30 minutes, and radioactivity was measured in a scintillation counter. Radiolabeled chylomicrons were generated from fasted *Gpihbp1*^−/−^ mice orally gavaged with olive oil mixed with [9,10-^3^H(N)]-Triolein (Perkin Elmer, NET431001MC) and [^4–14^C]-Cholesterol (Perkin Elmer, NEC018050UC). After 4 hours, mice were anesthetized, and blood was collected by cardiac puncture. Blood was diluted 1:10 with 0.5 M EDTA (pH 8.0) and centrifuged at 1,500×g for 15 min at 4°C to pellet blood cells. The plasma was then transferred to ultracentrifuge tubes and mixed 1:1 with PBS. After centrifugation at 424,000×g for 2 h at 10°C, the chylomicrons formed an upper layer. The chylomicron layer was resuspended in fresh PBS, and the centrifugation was repeated. Following the second centrifugation, the chylomicron layer was resuspended in PBS to the original plasma volume.

Clearance of DiI-labeled VLDL was carried out similarly using VLDL (density <1.019 g/mL) prepared from high-fat-, high-sucrose-fed *Ldlr*^−/−^ mice.

### Isolation of mouse LDL and TRL/RLP.

LDL (density 1.019–1.08 g/mL) and TRL/RLP (density <1.019 g/mL) were isolated by sequential ultracentrifugation from rapidly thawed plasma from 8 mice in each group (a total of 6 groups, diabetic and non-diabetic mice treated with cASO, APOC3 ASO or ANGPTL3 ASO, respectively). One hundred (100) μL plasma was added to 52.4 μL 1.063 g/mL KBr and 77.6 μL normal saline in a 230 μL centrifuge tube. The samples were spun in a TLA 100 rotor at 100,000 RPM (436,000×g) for 16.5 hours at 4°C. The top 75 μL was collected as the TRL/RLP fraction (<1.019 g/mL). The density of the bottom portion was measured, and the volume was brought to 230 μL with KBr solution to a final density of 1.08 g/mL. The samples were spun in a TLA 100 rotor at 100,000 RPM (436,000×g) for 4 hours at 4°C. The top 75 μL was collected as the LDL fraction (1.019–1.08 g/mL). Both LDL and TRL fractions were dialyzed against 20 mM potassium phosphate (pH 7.4 with 100 μM DTPA) before digestion and mass spectrometry analysis of apolipoproteins.

### Digestion of LDL and TRL/RLP fractions.

Sixteen μg of LDL and TRL was reduced with dithiothreitol and then alkylated with iodoacetamide in 0.5% sodium deoxycholate (SDC), 5% acetonitrile and 100 mM ammonium bicarbonate (NH_4_HCO_3_). ^15^N-labeled APOA1 (0.5 μg) was added to each sample before digestion as the internal standard. The LDL and TRL proteins were then incubated overnight (18 h) at 37°C with 20:1 (w/w, protein/enzyme) of sequencing grade modified trypsin. Eight (8) μL of 20% trifluoroacetic acid was added to the reaction mixture (to pH 2–3) to stop the digestion and precipitate the SDC. The SDC precipitation was spun down in an Eppendorf centrifuge 5810R at 4000 rpm for 45 min. Sixty μL of the supernatant (~6 μg of protein digest) was transferred to 600 μL Eppendorf tubes and kept at −80°C until mass spectrometry analysis.

### Liquid chromatography-electrospray ionization tandem mass spectrometric (LC-ESI-MS/MS) analysis of apolipoproteins in LDL and TRL/RLP by parallel reaction monitoring (PRM).

We used targeted proteomics with PRM to quantify 6 apolipoproteins in LDL and TRL, as previously reported ^31,40^. Briefly, LC-ESI-MS/MS analyses were performed in the positive ion mode with an ultrahigh-resolution accurate mass Orbitrap Fusion Lumos Tribrid Mass Spectrometer (Thermo Fisher Scientific, San Jose, CA) coupled to a nano-flow UHPLC system (EASY-nLC^™^ 1200 System, Thermo Fisher Scientific). LDL and TRL protein digests (equivalent to 0.4 μg of protein) were loaded onto a C-18 trap column (0.1 × 40 mm) at a flow rate of 1.2 μL/min for 10 minutes using 0.1% formic acid in water (solvent A). They were then separated at a flow rate of 0.3 μL/min, using a C-18 analytical column (0.1 × 200 mm). Both the trap and analytical columns were packed in-house with Magic C-18 reverse-phase resin (5 μm; 100 Å; Michrom Bioresources). A multistep gradient of solvent A and 0.1% formic acid in 80% acetonitrile in water (solvent B) was used for the separation. The columns were kept at room temperature, and the peptides were separated using the following gradient: 1% to 8% solvent B for 1 min; 8% to 40% solvent B for 18 min; 40% to 75% solvent B for 8 min; and 75% to 95% solvent B for 3 min. The column was subsequently washed for 2 min in 95% solvent B, 95% solvent B to 1% solvent B in 1 min, and re-equilibrated in 1% solvent B for 12 min. The mass spectrometer was operated in PRM mode.

For PRM analyses, a spray voltage of 2,100 V and an ion transfer tube temperature of 300°C were used for ionization. The acquisition method combined a full scan (MS1) method with a time-scheduled PRM (MS2) method. The retention time (RT) of each targeted peptide was determined in preliminary PRM test runs, and the scheduled time window was set to RT ± 1.5 min. For the full scan, a scan range of m/z 300–1600, an orbitrap resolution of 120,000, a target automatic gain control value of 400,000, and a maximum injection time of 100 milliseconds were used. For the PRM scan, a targeted list of precursor ions with charge state of +1, +2, or +3, depending on the peptide, were isolated using a 1.6 units of m/z window and an orbitrap resolution of 30,000; a target automatic gain control value of 50,000, and a maximum injection time of 54 milliseconds were used. Fragmentation was performed with normalized HCD collision energy of 30% and MS/MS scans were acquired with a starting mass of m/z 100, the ending mass being automatically defined by the m/z and the charge state of the precursor ion.

Candidate peptides for each protein were initially selected from the detected peptides by shotgun analysis. All peptides selected for a protein were unique to that protein. All selected peptides were then tested by PRM test runs, and finally, two or more peptides were measured for 5 apolipoproteins and one peptide for APOC2 (Supplemental Materials).

### Quantifying LDL and TRL proteins with ^15^N-labeled APOA1.

The relative levels of proteins in LDL or TRL were quantified by PRM using [^15^N]APOA1 as the internal standard^40^. The PRM data were analyzed with Skyline (version 24.1.0.199), an open-source program^41^. An equal amount of ^15^N-labeled APOA1 (1:32 ^15^N-APOA1to total protein in LDL or TRL) was added to TRL and LDL isolated from each mouse prior to digestion. The peak areas of all the transitions of a peptide detected by PRM analysis were summed to get the total peak area for the peptide. Transitions with peak interferences (e.g., the peak distorted by an interfering element) were not included. To normalize the peak area of a peptide, the total peak area of all selected transitions of the peptide was divided by the peak area of one peptide (^15^N-DYVSQFEGSALGK) from ^15^N-APOA1 and the ratios were used for quantification. To calculate the relative levels of peptides between the control and diabetic groups or among the different treatment groups, we set the average ratio of the peptide in non-diabetic mice treated with cASO to an arbitrary value of one. To obtain the relative level of a protein in LDL or TRL, if two or more peptides were quantified, the relative levels of the two or more peptides from each protein were averaged.

### Preparing aortas for scRNA-seq and flow cytometry.

Aortic arches (2–3 aortic arches/biological replicate, 3 biological replicates/group) from the heart to 2 mm past the left subclavian artery (no branches) were pooled for each N from STZ-diabetic mice and non-diabetic littermate controls 9 weeks after diabetes induction, and from LCMV-diabetic mice and non-diabetic littermates treated with cASO and LCMV-diabetic littermate mice treated with APOC3 ASO 12 weeks after diabetes induction. The arches were cleaned to remove adventitial adipose tissue and digested for 1 hour at 37 °C in Liberase TM (4 U/mL), hyaluronidase (60 U/mL), and DNase 1 (60 U/mL) in the presence of actinomycin (1 μg/mL), followed by sorting for DRAQ^+^ (nucleated), DAPI^−^ (live) cells. 20–30 000 cells were loaded into the Gel Bead-in Emulsion using the 10x Genomics Chromium Single Cell 3' Reagent Kits (v3.1 Chemistry Dual Index), according to the manufacturer’s instructions. 6,000 to 10,000 cells per sample and 25,000 reads per cell were targeted, which resulted in 12 cycles being used for cDNA amplification and sample index PCR. Sequencing was performed on an Illumina instrument using a NextSeq 2000 P3 Reagent (100 cycles).

For experiments where aortas were used for flow cytometry, aortas were cleaned and digested into single cells, as above, followed by staining using a live/dead viability dye and antibodies to CD45, CD11B, CD11C, F4/80, Ly6C, and LYVE1. Following staining and fixation, the cells were stained with BODIPY (ThermoFisher). For an example of gating, see Extended Data Fig. 8a. All gates were set based on flow minus one control.

### Single-cell RNA-sequencing.

The FASTQ files from each sample were processed by Cell Ranger v.7.1.0, where reads were aligned to mouse transcriptome reference mm10 2020-A. A unique molecular identifier (UMI) count matrix was obtained from each sample. The datasets were analyzed using Seurat v4.3.0 in R v4.2.0. Cells with less than 5% mitochondrial genes were maintained from each dataset. The cells with 200 to 4,000 genes were maintained from the STZ diabetes dataset and cells with >200 genes and <25,000 counts were maintained from the LCMV diabetes dataset. Raw UMI counts from each cell were normalized by the total expression in that cell, multiplied by 10,000, and then the natural log transformed the result by adding a pseudo-count of 1. Highly variable genes from each sample are based on the mean-variance relationship. We utilized the integration pipeline in Seurat to integrate datasets and minimize the batch effect. Integration anchors were identified based on 2,000 highly variable genes across datasets. Integration was then performed using the anchors identified. Two-dimensional UMAP was used for visualization using the first 40 principal components (PC) built on the integration assay. Graph-based clustering was performed on the integrated dataset. A Shared Nearest Neighbor (SNN) graph was constructed with 40 PCs as input, and the clusters were identified using a resolution parameter of 0.8 for the STZ dataset and 0.2 for the LCMV dataset. Unsupervised clusters were annotated to different cell types based on best-matched annotation from previous literature^29^ using the SingleR function. The leukocyte cluster was reclustered and annotated against a publication by Zernecke et al.^30^ and visualized using the original UMAP. For the top genes in each cell type, see Supplemental Excel file 1. Differential expression genes (DEGs) between non-diabetic (ND) and diabetic (D) samples were calculated using the Wilcox test and Benjamini & Hochberg correction, with each gene required to be expressed in 10% of cells in either of the two groups. Genes with fold change ≥ 0.5 and adjusted P-value < 0.05 were considered differentially expressed. A complete list of DEG, GSEA, and results from IPA can be found in Supplemental Excel files 2–3. For the ingenuity pathway analysis (IPA) and upstream regulator analysis, only Z scores of 2 or greater (positive or negative) were considered. The data were then ranked by statistical significance (p<0.05 for IPA and adjusted p<0.05 for upstream regulators) in the LCMV model, and only data passing these two parameters are included in the figures. All data passing the Z score cutoff are included in Supplemental Excel file 3.

### Real-time PCR.

Gene expression in the liver was quantified by real-time PCR. RNA isolation and the real-time PCR protocol were performed as described previously^42^. RNA was isolated using Nucleospin RNA Plus kits according to the manufacturer’s protocols. Real-time PCR was performed using the SYBR Green 1 detection method (ThermoScientific). Cycle threshold (Ct) values were normalized to *Rn18s* and presented as fold-over control. Primer sequences are listed in Key Reagents.

### RNA-sequencing of human SMCs stimulated with delipidated human APOC3.

Human primary aortic smooth muscle cells (SMCs) were isolated from a deceased newborn donor (male sex based on the expression of *DDX3Y*, *UTY,* and *KDM5D* and very low to non-detectable expression of *XIST*) and cultured in 10% FBS in high glucose DMEM. After the SMCs (passage 3 at 80% confluency in a 24-well plate) were preincubated in low-serum medium (0.5% FBS in high glucose DMEM) for 24 h, APOC3 isolated from human plasma (MyBiosource, catalog #MBS135971 lot #47644 1.5 mg/mL in 10 mM NH_4_HCO_3_, pH 7.4) was added to the cells at a final concentration of 40 μg/mL in the low-serum medium for 24 h. Control cells received vehicle (NH_4_HCO_3_). Endotoxin levels at the final concentration of APOC3 used were <4 mEU/mL (Pierce^™^ Chromogenic Endotoxin Quant Kit, A39552, ThermoFisher). At the end of treatment, the cells were washed and lysed in a lysis buffer for extraction of RNA by using a Quick RNA kit (R1053, Zymo Research). RNA sequencing was performed in the Genomics core at Benaroya Research Institute, Seattle, WA (Supplemental Excel 4).

### Sex as a biological variable.

Both sexes were analyzed, as indicated in the figure legends and tables.

### Statistical analysis.

**Figure 1.**
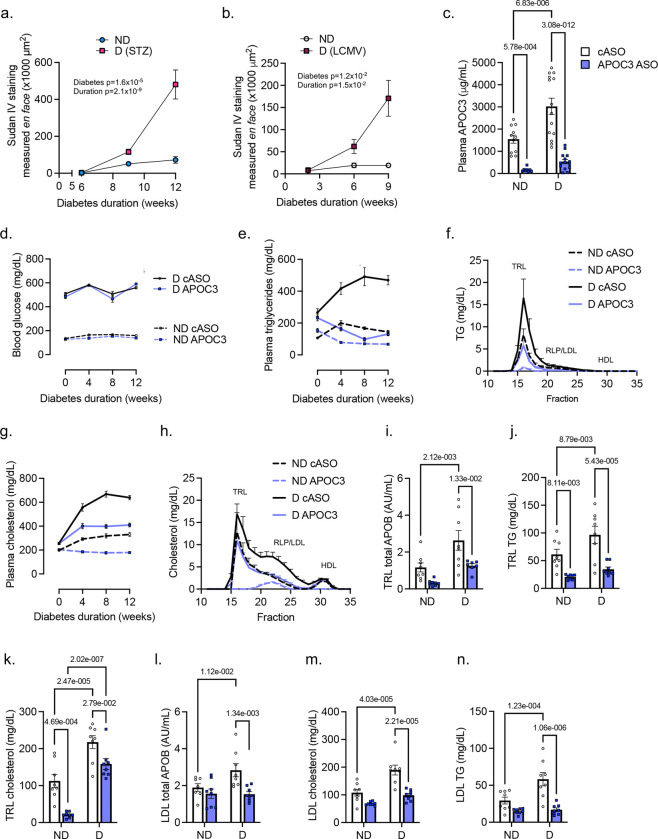
Hepatic APOC3 silencing prevents elevated plasma levels of TRL/RLP and LDL in diabetes. Aortic lipophilic area at indicated time points in mouse models of STZ-diabetes (**a**) and LCMV-induced diabetes (**b**) fed a low-fat semipurified diet at the onset of diabetes (week 0). **c-n.** Diabetic (D, LCMV model) and non-diabetic (ND) littermates were injected weekly with control GalNAc ASO (cASO) or APOC3 GalNAc ASO. **c**. Plasma APOC3 at 12 weeks. **d**. Blood glucose over 12-weeks. **e-h**. Plasma triglycerides (TG), cholesterol-, and triglyceride- and cholesterol lipoprotein profiles. **i-k**. Isolated TRLs analyzed for APOB content, triglycerides, and cholesterol. **l-n**. Isolated LDL analyzed for APOB content, cholesterol, and triglycerides. Data are expressed as mean ± SEM, two-way ANOVA (see Original Data supplement for details on number of mice/group and statistical analyses).

**Figure 2.**
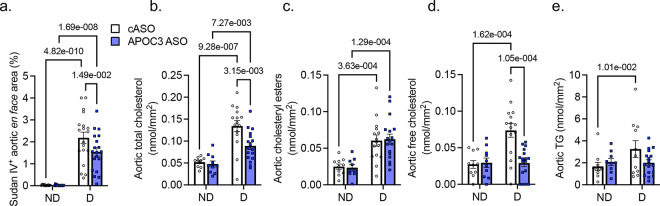
Hepatic APOC3 silencing prevents elevated levels of aortic free cholesterol in diabetes. Diabetic (D, LCMV model) and non-diabetic (ND) littermates were injected weekly with control GalNAc ASO (cASO) or APOC3 GalNAc ASO. **a**. Aortic Sudan IV-positive lipophilic *en face* area at the end of the 12-week study. **b**. Aortic total cholesterol. **c**. Aortic cholesteryl esters (CE). **d**. Aortic free cholesterol. **e**. Aortic triglycerides (TG). Data are expressed as mean ± SEM, two-way ANOVA (see Original Data supplement for details on number of mice/group and statistical analyses).

Statistical analyses were performed on distinct samples using GraphPad Prism 10.3 (La Jolla, CA). D'Agostino-Pearson omnibus normality tests were performed to evaluate if the data were normally distributed. Statistical outliers were identified by the ROUT (Q=0.1%) method. Statistical analyses and statistical outliers are indicated in the Original Data file. We used two-tailed unpaired Student’s *t*-tests for normally distributed data and Mann-Whitney tests for non-normally distributed data to compare differences when only two groups were compared. To compare more groups, we used a two-way ANOVA. If there were two subgroups (non-diabetic and diabetic), Tukey’s multiple comparisons tests or Šídák's multiple comparisons tests were used comparing within each subgroup and across the same treatment group (e.g., ND cASO vs. D cASO, D cASO vs D APOC3 ASO). For data presented in Fig. 1–2 and Extended Fig. 4–6, the statistical analyses are based on the 6 group comparisons.

### Study approvals.

Institutional review boards at each of the six MESA study sites approved the study protocol and written informed consent was obtained from all study participants. Isolation of aortic human SMCs was approved on the University of Washington’s Institutional Review Board protocol STUDY00008441. All mouse experiments were performed in accordance with an approved University of Washington Institutional Animal Care and Use Committee protocol (protocol 3154–01). This paper has been reviewed and approved by the MESA Publications and Presentations Committee.

## Supplementary Material

Supplementary Files

This is a list of supplementary files associated with this preprint. Click to download.

• ExtendedDataFigures.pdf

• Resourceinformation.docx

• nreditorialpolicychecklistKanter.pdf

• SupplementalExcel1ClusterMarkers.xlsx

• SupplementalExcel3IPA.xlsx

• OriginalDataKanter.xlsx

• nrreportingsummaryKanter.pdf

• SupplementalExcel4SMCAPOC3.xlsx

• SupplementalExcel2DEG.xlsx

## Figures and Tables

**Figure 3.**
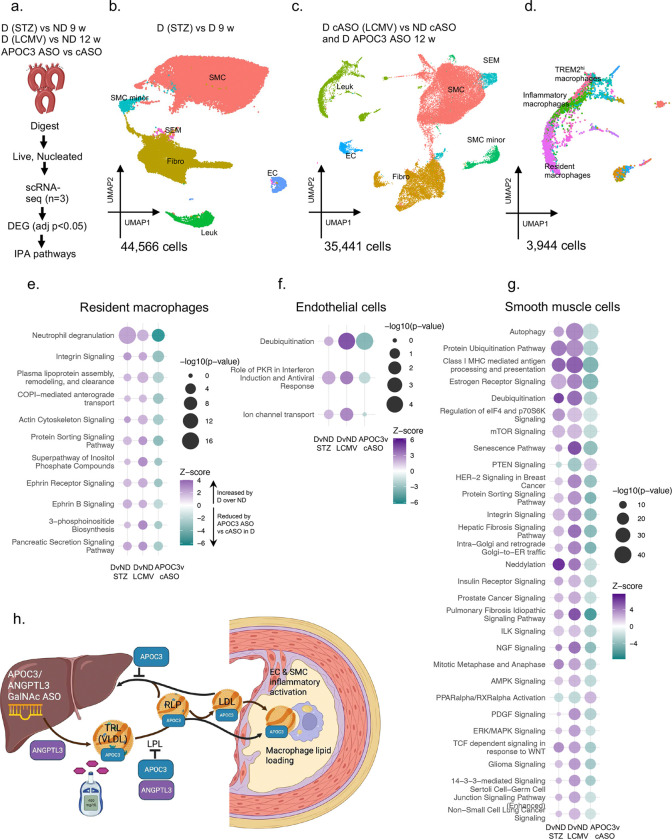
A majority of diabetes-induced transcriptional signatures in arterial cells are mediated by hepatic APOC3. **a.** Lesioned aortic arches from 3 biological replicates from the two diabetes models and cASO- and APOC3 ASO-treated diabetic (D) mice and non-diabetic (ND) cASO controls analyzed by scRNA-seq. **b-c.** UMAPs of identified cells. **d.** UMAP of reclustered leukocytes in the LCMV diabetes model. **e-g.** IPA using DEGs (adjusted p<0.05) in (**e**) the resident macrophage cluster (**f**) EC cluster and (**g**) SMC cluster from D (STZ) versus ND, D (LCMV) versus ND, and APOC3 ASO (LCMV D) versus cASO (LCMV D). The top 30 pathways based on Z-scores and p-values are shown in g. **h.** Schematic representation of the effects of hepatic APOC3 and ANGPTL3 silencing.

**Table 1:** Association of plasma APOC3 concentrations with incident CVD (myocardial infarction, resuscitated cardiac arrest or stroke) in Multiethnic Study of Atherosclerosis (MESA)

Group	N	Women	APOC3 (mg/dL)	No. of CVD Events	Median follow-up (yrs)	HR per 1 SD APOC3 (95% CI)*
All	5,743	52%	9.3 ± 3.9	670	15.6	1.06 (0.98, 1.15)
Normal FG	4,187	55%	9.1 ± 3.8	414	15.7	0.98 (0.89, 1.08)
Impaired FG	822	44%	9.5 ± 3.9	102	15.5	1.09 (0.88, 1.34)
Diabetes	724	47%	9.9 ± 4.2	153	14.1	1.21 (1.01, 1.45)

* Unadjusted Cox proportional hazard regression models; p=0.038 for interaction between APOC3 and fasting glucose (FG) status. Diabetes was defined as FG ≥ 7.0 mmol/L (126 mg/dL) or self-reported use of hypoglycemic drugs. Impaired fasting glucose was defined as FG 5.6 – 6.9 mmol/L (100 – 125 mg/dL) and normal FG reflected fasting values below 5.6 mmol/L (100 mg/dL).

## Data Availability

The Original Data file reports values for all data points in graphs on mouse experiments. The data files containing the DEGs and the results from the GSEA and IPA are in Supplemental Excel files 1–4. The datasets generated and analyzed during the current study are available in the National Center for Biotechnology Information Gene Expression Omnibus (GEO) repository (GSE “pending”). The human datasets can be accessed by reasonable request to the MESA Publication and Presentations Committee (https://www.mesa-nhlbi.org).

## References

[1] Bergmark B.A., Olezarsen for Hypertriglyceridemia in Patients at High Cardiovascular Risk. N Engl J Med 390, 1770–1780 (2024). 38587249 10.1056/NEJMoa2402309

[2] Ballantyne C.M., Plozasiran, an RNA Interference Agent Targeting APOC3, for Mixed Hyperlipidemia. N Engl J Med 391, 899–912 (2024). 38804517 10.1056/NEJMoa2404143

[3] Ballantyne C.M., Effect of Targeting apoC-III with Plozasiran on Lipoprotein Particle Size and Number in Hypertriglyceridemia. J Am Coll Cardiol (2025). 10.1016/j.jacc.2025.03.496 40099777

[4] Rosenson R.S., Zodasiran, an RNAi Therapeutic Targeting ANGPTL3, for Mixed Hyperlipidemia. N Engl J Med 391, 913–925 (2024). 38809174 10.1056/NEJMoa2404147

[5] Pollin T.I., A null mutation in human APOC3 confers a favorable plasma lipid profile and apparent cardioprotection. Science 322, 1702–1705 (2008). 19074352 10.1126/science.1161524 PMC2673993

[6] Tg, Loss-of-function mutations in APOC3, triglycerides, and coronary disease. N Engl J Med 371, 22–31 (2014). 24941081 10.1056/NEJMoa1307095 PMC4180269

[7] Jorgensen A.B., Frikke-Schmidt R., Nordestgaard B.G. & Tybjaerg-Hansen A. Loss-of-function mutations in APOC3 and risk of ischemic vascular disease. N Engl J Med 371, 32–41 (2014). 24941082 10.1056/NEJMoa1308027

[8] Stitziel N.O., ANGPTL3 Deficiency and Protection Against Coronary Artery Disease. J Am Coll Cardiol 69, 2054–2063 (2017). 28385496 10.1016/j.jacc.2017.02.030 PMC5404817

[9] Landfors F., Henneman P., Chorell E., Nilsson S.K. & Kersten S. Drug-target Mendelian randomization analysis supports lowering plasma ANGPTL3, ANGPTL4, and APOC3 levels as strategies for reducing cardiovascular disease risk. Eur Heart J Open 4, oeae035 (2024). 38895109 10.1093/ehjopen/oeae035 PMC11182694

[10] Gordts P.L., ApoC-III inhibits clearance of triglyceride-rich lipoproteins through LDL family receptors. J Clin Invest 126, 2855–2866 (2016). 27400128 10.1172/JCI86610 PMC4966320

[11] Shimamura M., Angiopoietin-like protein3 regulates plasma HDL cholesterol through suppression of endothelial lipase. Arterioscler Thromb Vasc Biol 27, 366–372 (2007). 17110602 10.1161/01.ATV.0000252827.51626.89

[12] Wu L., Soundarapandian M.M., Castoreno A.B., Millar J.S. & Rader D.J. LDL-Cholesterol Reduction by ANGPTL3 Inhibition in Mice Is Dependent on Endothelial Lipase. Circ Res 127, 1112–1114 (2020). 32808882 10.1161/CIRCRESAHA.120.317128 PMC10150441

[13] Fappi A, Effect of complete, lifelong ANGPTL3 deficiency on triglyceride-rich lipoprotein kinetics. Cell Rep Med Online ahead of print, May 29(2025). 10.1016/j.xcrm.2025.102152 PMC12208341 40446802

[14] Ginsberg H.N. & Goldberg I.J. Broadening the Scope of Dyslipidemia Therapy by Targeting APOC3 (Apolipoprotein C3) and ANGPTL3 (Angiopoietin-Like Protein 3). Arterioscler Thromb Vasc Biol 43, 388–398 (2023). 36579649 10.1161/ATVBAHA.122.317966 PMC9975058

[15] Bjornson E., Quantifying Triglyceride-Rich Lipoprotein Atherogenicity, Associations With Inflammation, and Implications for Risk Assessment Using Non-HDL Cholesterol. J Am Coll Cardiol 84, 1328–1338 (2024). 39322327 10.1016/j.jacc.2024.07.034 PMC7616757

[16] Chait A., Remnants of the Triglyceride-Rich Lipoproteins, Diabetes, and Cardiovascular Disease. Diabetes 69, 508–516 (2020). 32198194 10.2337/dbi19-0007 PMC7085249

[17] Izquierdo M.C., Hyperchylomicronemia causes endothelial cell inflammation and increases atherosclerosis. Res Sq (2024).

[18] Tall A.R., Thomas D.G., Gonzalez-Cabodevilla A.G. & Goldberg I.J. Addressing dyslipidemic risk beyond LDL-cholesterol. J Clin Invest 132(2022). 10.1172/JCI148559 PMC8718149 34981790

[19] Ginsberg H.N. Lipoprotein physiology in nondiabetic and diabetic states. Relationship to atherogenesis. Diabetes Care 14, 839–855 (1991). 1959476 10.2337/diacare.14.9.839

[20] Eckel R.H., Bornfeldt K.E. & Goldberg I.J. Cardiovascular disease in diabetes, beyond glucose. Cell Metab 33, 1519–1545 (2021). 34289375 10.1016/j.cmet.2021.07.001 PMC8411849

[21] Kanter J.E., Increased apolipoprotein C3 drives cardiovascular risk in type 1 diabetes. J Clin Invest 129, 4165–4179 (2019). 31295146 10.1172/JCI127308 PMC6763229

[22] Basu A., Serum apolipoproteins and apolipoprotein-defined lipoprotein subclasses: a hypothesis-generating prospective study of cardiovascular events in T1D. J Lipid Res 60, 1432–1439 (2019). 31203233 10.1194/jlr.P090647 PMC6672041

[23] Jansson Sigfrids F., Apolipoprotein C-III predicts cardiovascular events and mortality in individuals with type 1 diabetes and albuminuria. J Intern Med 291, 338–349 (2022). 34817888 10.1111/joim.13412 PMC9298713

[24] Colombo M., Apolipoprotein CIII and N-terminal prohormone b-type natriuretic peptide as independent predictors for cardiovascular disease in type 2 diabetes. Atherosclerosis 274, 182–190 (2018). 29793175 10.1016/j.atherosclerosis.2018.05.014

[25] Cervantes J., Elevated apolipoprotein C3 augments diabetic kidney disease and associated atherosclerosis in type 2 diabetes. JCI Insight 9(2024). 10.1172/jci.insight.177268 PMC11383354 38743496

[26] Malick W.A., Clinical Trial Design for Triglyceride-Rich Lipoprotein-Lowering Therapies: JACC Focus Seminar 3/3. J Am Coll Cardiol 81, 1646–1658 (2023). 37076219 10.1016/j.jacc.2023.02.034

[27] Shimizu-Albergine M., CREBH normalizes dyslipidemia and halts atherosclerosis in diabetes by decreasing circulating remnant lipoproteins. J Clin Invest 131(2021). 10.1172/JCI153285 PMC8592537 34491909

[28] Willecke F., Lipolysis, and not hepatic lipogenesis, is the primary modulator of triglyceride levels in streptozotocin-induced diabetic mice. Arterioscler Thromb Vasc Biol 35, 102–110 (2015). 25395613 10.1161/ATVBAHA.114.304615 PMC4270817

[29] Pan H., Single-Cell Genomics Reveals a Novel Cell State During Smooth Muscle Cell Phenotypic Switching and Potential Therapeutic Targets for Atherosclerosis in Mouse and Human. Circulation 142, 2060–2075 (2020). 32962412 10.1161/CIRCULATIONAHA.120.048378 PMC8104264

[30] Zernecke A., Integrated single-cell analysis-based classification of vascular mononuclear phagocytes in mouse and human atherosclerosis. Cardiovasc Res 119, 1676–1689 (2023). 36190844 10.1093/cvr/cvac161 PMC10325698

[31] Hsu C.C., Apolipoprotein C3 induces inflammasome activation only in its delipidated form. Nat Immunol 24, 408–411 (2023). 36781985 10.1038/s41590-023-01423-2 PMC9992333

[32] Duewell P., NLRP3 inflammasomes are required for atherogenesis and activated by cholesterol crystals. Nature 464, 1357–1361 (2010). 20428172 10.1038/nature08938 PMC2946640

[33] Ramms B., Interventional hepatic apoC-III knockdown improves atherosclerotic plaque stability and remodeling by triglyceride lowering. JCI Insight 7(2022). 10.1172/jci.insight.158414 PMC9310539 35653195

[34] Bild D.E., Multi-Ethnic Study of Atherosclerosis: Objectives and Design. American Journal of Epidemiology 156, 871–881 (2002). 12397006 10.1093/aje/kwf113

[35] Mendivil C.O., Zheng C., Furtado J., Lel J. & Sacks F.M. Metabolism of very-low-density lipoprotein and low-density lipoprotein containing apolipoprotein C-III and not other small apolipoproteins. Arterioscler Thromb Vasc Biol 30, 239–245 (2010). 19910636 10.1161/ATVBAHA.109.197830 PMC2818784

[36] Folsom A.R., Coronary artery calcification compared with carotid intima-media thickness in the prediction of cardiovascular disease incidence: the Multi-Ethnic Study of Atherosclerosis (MESA). Arch Intern Med 168, 1333–1339 (2008). 18574091 10.1001/archinte.168.12.1333 PMC2555989

[37] Renard C.B., Diabetes and diabetes-associated lipid abnormalities have distinct effects on initiation and progression of atherosclerotic lesions. J Clin Invest 114, 659–668 (2004). 15343384 10.1172/JCI17867 PMC514580

[38] Kothari V., Imbalance of APOB Lipoproteins and Large HDL in Type 1 Diabetes Drives Atherosclerosis. Circ Res 135, 335–349 (2024). 38828596 10.1161/CIRCRESAHA.123.323100 PMC11223987

[39] Vaisar T., High Concentration of Medium-Sized HDL Particles and Enrichment in HDL Paraoxonase 1 Associate With Protection From Vascular Complications in People With Long-standing Type 1 Diabetes. Diabetes Care 43, 178–186 (2020). 31597668 10.2337/dc19-0772 PMC6925582

[40] Shao B., Pulmonary surfactant protein B carried by HDL predicts incident CVD in patients with type 1 diabetes. J Lipid Res 63, 100196 (2022). 35300983 10.1016/j.jlr.2022.100196 PMC9010748

[41] MacLean B., Skyline: an open source document editor for creating and analyzing targeted proteomics experiments. Bioinformatics 26, 966–968 (2010). 20147306 10.1093/bioinformatics/btq054 PMC2844992

[42] Kanter J.E., Diabetes promotes an inflammatory macrophage phenotype and atherosclerosis through acyl-CoA synthetase 1. Proc Natl Acad Sci U S A 109, E715–724 (2012). 22308341 10.1073/pnas.1111600109 PMC3311324

